# A raw data on the physico-chemical water parameters and sedimentation rates of two different aquatic macrophytes in Tasik Berombak, Malaysia

**DOI:** 10.1016/j.dib.2023.109397

**Published:** 2023-07-09

**Authors:** Jia Yi Choe, Ku Mohd Kalkausar Ku Yusof, Shahrudin Rohani

**Affiliations:** Faculty of Science and Marine Environment, Universiti Malaysia Terengganu, Kuala Nerus 21030, Malaysia

**Keywords:** Correlation heatmap, Ecology alteration, Emergent macrophyte, Freshwater, Invasive aquatic plant, Land use changes

## Abstract

The excessive growth of aquatic macrophytes in a water system has a negative effect on the lake ecosystem. This article presents data on water parameters and sedimentation rates from sites that include different aquatic macrophytes at Tasik Berombak, a freshwater lake on Peninsular Malaysia's eastern coast. Areas with *Hanguana* *malayana* and *Pandanus helicopus* were selected for sampling, while an area without aquatic macrophytes served as the control. At the lake's surface and bottom, temperature, conductivity, total dissolved solids, dissolved oxygen (D.O.), and pH were measured *in situ*. The surface water was sampled for chemical analysis in the laboratory (chlorophyll-a, total suspended solids, total carbon, total organic carbon, inorganic carbon, total dissolved nitrogen, total dissolved phosphorus). Settling sediment was collected using cylinder traps deployed under the macrophytes at the bottom of the lake. The presented data includes the water parameters according to plant-base area, depth differentiation (top versus bottom), and variable correlation analysis. Understanding the impact of excessive aquatic plants on the lake ecosystem in a tropical environment requires information on water parameters and sedimentation rates from the aquatic plants. Therefore, these data can be used to monitor the impact of land use change on the aquatic plant community and, ultimately, the lake ecosystem.

**Specifications Table**

**Table:** 

Subject	Environmental Science
Specific subject area	Ecology, Hydrology and Water Quality
Type of data	Graph Figure
How the data were acquired	Data were acquired through *in situ* measurement and sampling, chemical analysis, and sediment trapping. 1. *In situ* measurement: Water depth was taken using SM-5 Portable Water Depth Sounder Gauge. Water temperature, conductivity, total dissolved solids (TDS), salinity, dissolved oxygen (DO), and pH on the water surface and bottom of the lake were acquired using YSI Handheld Multiparameter Instrument. 2. Chemical analysis of surface water samples collected includes: - Chlorophyll-a was measured using Shimadzu UV-1800 UV-Vis Spectrophotometer (Jeffrey & Humphrey's Trichromatic Method). - Total dissolved nitrogen (Modified APHA Method 4500-N) and total dissolved phosphorus (Modified APHA Method 4500-P) were measured using Shimadzu UV-1800 UV-Vis Spectrophotometer. - Total carbon, inorganic carbon and organic carbon were measured using Shimadzu TOC 5000 Analyzer (APHA Method 5310-B). - Total suspended solids were obtained through filtration and drying (APHA Method 2540-D). 3. Sediments were collected using sediment traps deployed on the lakebed.
Data format	Raw Analyzed
Description of data collection	Three sampling stations were established according to the different aquatic macrophytes species. During data collection, the in-situ measurement was done first. Sediment traps were installed at the bottom of the lake in each station. The sediments and surface water samples were collected and kept in an ice box before being transported to the lab.
Data source location	City / Town / Region: Setiu / Terengganu Country: Malaysia GPS coordinates of each sampling station: *Hanguana malayana*: 5°40′2″ N 102°41′20″ E *Pandanus helicopus*: 5°40′6″ N 102°41′13″ E Non-vegetated area: 5°40′5″ N 102°41′20″ E
Data accessibility	Repository name: Mendeley Data Data identification number: 10.17632/6dxbrrp5zp.1 Direct URL to data: https://data.mendeley.com/datasets/6dxbrrp5zp

## Value of the Data

• The data reflect the water quality analysis of the excessive growth of aquatic plants in the tropical climate. The data improve our understanding of the effect of the excessive growth of aquatic plants on the water parameters and sedimentation rate in the tropical lake.
• The data on water parameters can be used by the scientific community to compare the parameters across different lakes. This data can be used by the Terengganu State Park in its management plan as a baseline for future development.
• The information on the water parameters can be used to monitor the effects of land use changes such as agriculture and other anthropogenic activities in the lake's vicinity.
• Data on the sedimentation rate can be used to develop a model of the sedimentation process under different circumstances.

## 1 Objective

This dataset compares the water quality and sedimentation rates of two emergent macrophytes from a natural lake in Peninsular Malaysia. The objective of generating this dataset was to better understand how the excessive growth of aquatic vegetation in the tropical environment affected the abiotic lake variables. This dataset could be utilised to understand the impact of human activities on the health of lake ecosystems.

## 2 Data Description

**Fig. 1:**
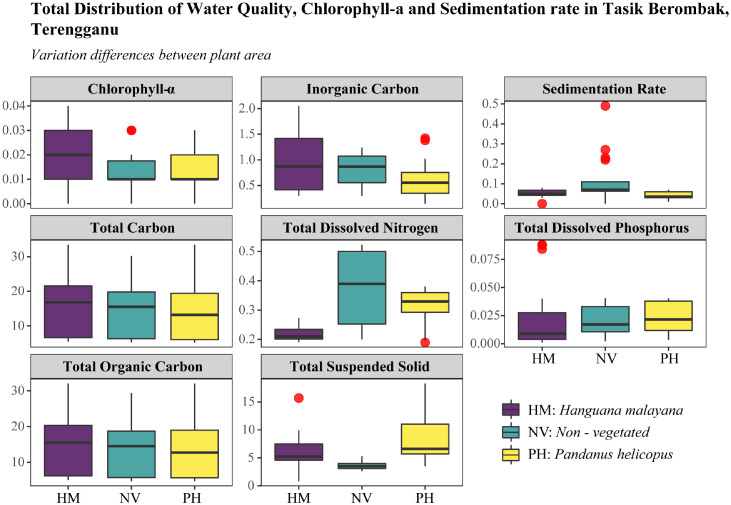
The total measurement of several water quality variables, chlorophyll-a, and sedimentation rate, in three plant types (*Hanguana malayana*, non-vegetated, and *Pandanus helicopus*).

The datasets containing seven subfigures were clustered into three major groups representing the water quality and sedimentation rate based on the plant-based area, depth differentiation (top versus bottom), and correlation analysis between variables. Fig. 1 displays the distribution variety of Carbon, Nitrogen, Phosphorus, and Chlorophyll-a, and the sedimentation rate according to the plant-based area. The variation was observed in two distinct plant species, *Hanguana malayana,* and *Pandanus helicopus*. A non–vegetated area was designated as a control for comparing the differences between the two plant species. The measurement in Fig. 1 was derived solely from measurements of surface water since the depth is not that much different. According to Fig. 1, *Hanguana malayana* had higher concentrations of chlorophyll-a and the carbon group (total carbon, total organic carbon, and inorganic carbon). Non–vegetated areas had the highest concentrations of total dissolved nitrogen and sedimentation rate, while *Pandanus helicopus* had the highest concentrations of total dissolved phosphorus and total suspended solids.

**Fig. 2:**
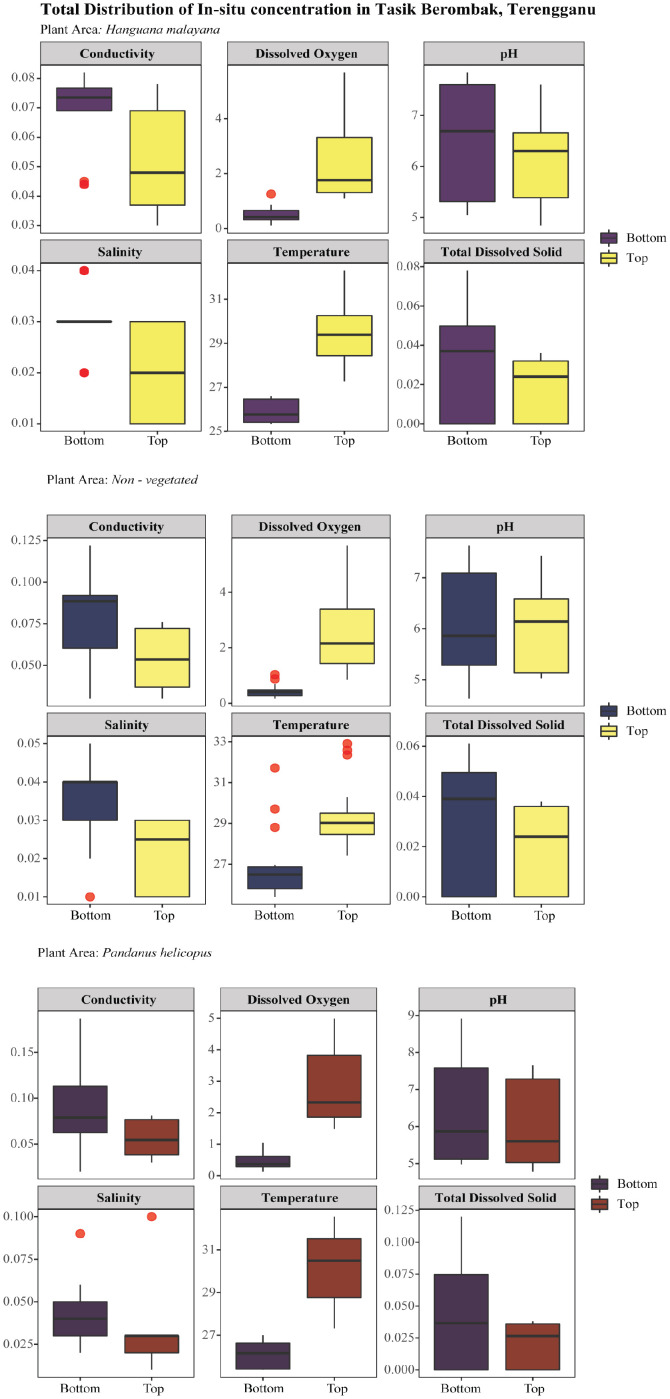
The box – whisker plots comparing the bottom and top *in-situ* results in three plant areas (*Hanguana malayana*, non-vegetated, and *Pandanus helicopus*).

Fig. 2 illustrates the total distribution of *in-situ* results, which is divided into three groups: *Hanguana malayana* (purple/yellow) on the top, non-vegetated (blue/yellow) in the middle, and *Pandanus helicopus* (purple/red) at the bottom of the figure. To comprehend this, the explanation in this section was divided into two simple types. The *in-situ* variation result was first explained between the bottom and then between the top (surface) readings. The non–vegetated area had the highest average temperature profile at the bottom, with 26.88 ± 1.63˚C. The *Pandanus helicopus* plant exhibited the highest conductivity (0.08 ± 0.04 µS/cm), total dissolved solids (0.06 ± 0.028 mg/L), and salinity (0.04 ± 0.02 ppt) measurements. *Hanguana malayana* had the highest average concentrations of dissolved oxygen (0.49 ± 0.30 mg/L) and pH (6.43 ± 1.10). For the top (surface) readings, *Pandanus helicopus* has the highest average *in-situ* results for temperature (30.21 ± 1.64˚C), conductivity (0.05 ± 0.02 µS/cm), total dissolved solids (0.03 ± 0.006 mg/L), dissolved oxygen (2.79 ± 1.20 mg/L), and salinity (0.03 ± 0.02 ppt) compared to the non-vegetated and *Hanguana malayana*. The location of the *Hanguana malayana* is where the maximum pH reading was recorded (6.20 ± 0.89).

**Fig. 3:**
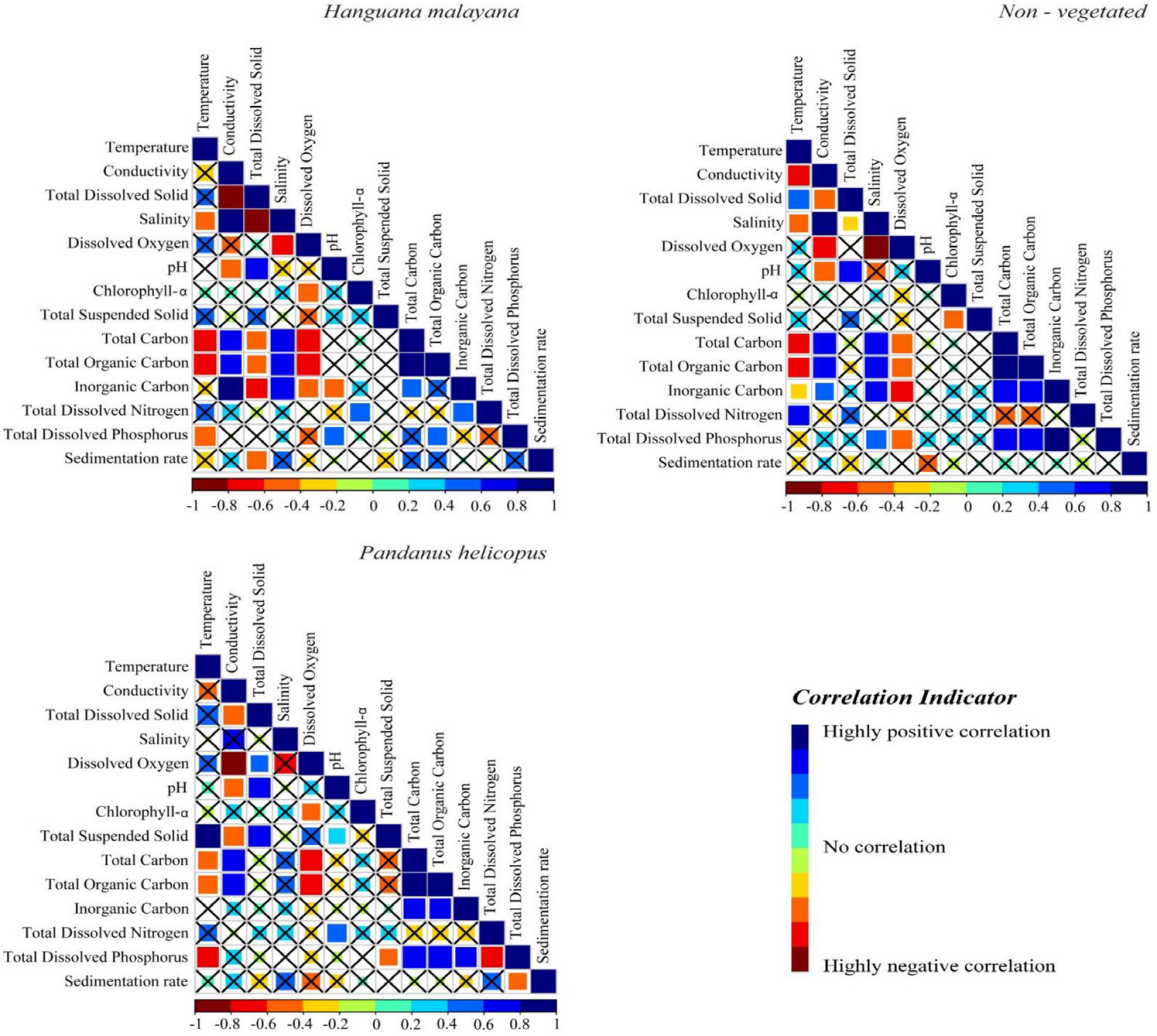
Heatmap showing the correlation between three different plant types (*Hanguana malayana, non*-vegetated, and *Pandanus helicopus*).

Lastly, Fig. 3 exhibits the monotonous correlation between variables involved in this study for three different plants. The correlation integrated the spearman's rank correlation with a 95% confidence interval (p <0.05). All “X” in the correlation heatmap are denoted with an insignificant correlation.

## 3 Experimental Design, Materials and Methods

Three sampling stations were established according to the dominant plant species; Station 1 represented the population of *Hanguana malayana*; Station 2 represented the population of *Pandanus helicopus*; Station 3 was the control, which is an area without vegetation. Three sets of sediment traps were installed at each station's bottom of the water body. The traps were left in place and collection was made during the subsequent sampling. Raw water samples were taken from the installed sites during the sediment collection.

### 3.1 *In Situ* Physical Parameters

Physical parameters such as Temperature (°C), Conductivity (ms/cm), Total Dissolved Solids (TDS) (g/L), Salinity (ppt), Dissolved Oxygen (DO) (mg/L), and pH were measured using a YSI Handheld Multiparameter Instrument on both the water surface and lake bottom. The lake's water depth was measured using the SM-5 Portable Water Depth Sounder Gauge.

### 3.2 Water Sample Collection, Storing And Processing

Surface water samples were collected using opaque HDPE bottles at each site and immediately placed in an ice-filled ice box before being transferred back to the laboratory. All water samples were frozen at -20°C until filtration. Water samples were vacuum filtered using 0.7 µm, 47 mm diameter Whatman 1825-047 GF/F filter paper that was pre-baked at 500°C for 4 h. Residues on the filter paper were used for determining the Total Suspended Solids (TSS) and Chlorophyll-a. The filtrate was kept separately for carbon content and nutrient analysis before refrigerating at 4°C. Filtrate for carbon content analysis was acidified to pH 2 for preservation.

### 3.3 Chlorophyll-a Determination

Filter papers with residue were placed into a 50 mL centrifuge tube for extraction by adding 5 mL of 90% acetone. The tubes were centrifuged at 4000 rpm, 4°C, for 20 min. Separated solutions were read using a Shimadzu UV-1800 UV-Vis Spectrophotometer at 750 nm, 664 nm, 647 nm and 630 nm absorbance to obtain the values for chlorophyll-a concentration [1,2].

### 3.4 Total Carbon, Total Organic Carbon, And Inorganic Carbon

Refrigerated samples were transferred into glass vials for detection using a Shimadzu TOC analyzer. Total organic carbon values were obtained by deducing the values of inorganic carbon from total carbon [3].

### 3.5 Total Dissolved Nitrogen

10 mL of filtrate and 5 mL of persulfate oxidizing agent were placed into a centrifuge tube and autoclaved at 120°C for 55 min. After being cooled to room temperature, the solutions were filtered through Whatman 0.45 µm pore filter paper. 4 mL of filtered samples were placed into an empty centrifuge tube and spiked with 1 mL of mixed reagent of Sulphanilamide, N-(1-naphthyl)-ethylenediamine dihydrochloride (NED), vanadium (III) chloride and deionized water. Both solutions were mixed well, placed in a 60°C water bath for 25 min for reduction, and let cool to room temperature. The samples were then read at 540 nm absorbance using a UV-Vis Spectrophotometer with a series of nitrate standards and deionized water as a blank [3].

### 3.6 Total Dissolved Phosphorus

20 mL of filtrate and 10 mL of persulfate oxidizing agent were placed into a centrifuge tube and autoclaved at 120°C for 55 min. After being cooled to room temperature, the solution was filtered through Whatman 0.45 µm pore filter paper. 20 mL of filtered samples were placed into an empty centrifuge tube and spiked with 2 mL of a combined reagent of sulphuric acid, potassium antimonyl tartrate solution, ammonium molybdate solution, and ascorbic acid. Both solutions were thoroughly mixed and let to sit for 10 min. The samples were then read at 880 nm absorbance using a UV-Vis Spectrophotometer with a series of phosphorus standards and deionized water as a blank [3].

### 3.7 Total Suspended Solids

1 L of water samples were filtered through pre-weighed Whatman GF/F filter paper and dried in the oven at 100°C until a constant weight was obtained. The total suspended solids value was obtained by substituting values in the formula below [3]:

$$\text{TSS}\;\text{(mg/L)}=\frac{(\mathrm{final}\;\mathrm{weight}(g)-\mathrm{initial}\;\mathrm{weight}(g))\;x\;1000}{\mathrm{sample}\;\mathrm{volume}\;\mathrm{in}\;L}$$

### 3.8 Sediment Setting Experiment

The sediment settling experiment was done by deploying a sediment trap, which comprises 4 PVC tubes secured firmly in a stainless-steel frame with cable ties. The volume of each PVC tube is approximately 1,413.72 m^3^. Sediment traps were retrieved after two months, and the content accumulated in the traps was transferred into a 5 L HDPE bottle to be returned to the laboratory. The sediment in the bottle was then transferred into a measuring cylinder and left to settle for a week. The settled sediments were dried at 60°C, and weights were recorded. The sedimentation rate was expressed as g/m^2^/day.

## Ethics Statements

This research does not involve any humans or animals.

## CRediT authorship contribution statement

**Jia Yi Choe:** Investigation, Writing – original draft. **Ku Mohd Kalkausar Ku Yusof:** Formal analysis, Visualization, Writing – original draft. **Shahrudin Rohani:** Conceptualization, Methodology, Funding acquisition, Writing – review & editing.

## Declaration of Competing Interest

The authors declare that they have no known competing financial interests or personal relationships that could have appeared to influence the work reported in this paper.

## Data Availability

Dataset Tasik Berombak (Original data) (Mendeley Data).
